# Genome-Wide Association Study Reveals Candidate Genes for Growth Relevant Traits in Pigs

**DOI:** 10.3389/fgene.2019.00302

**Published:** 2019-04-05

**Authors:** Zhenshuang Tang, Jingya Xu, Lilin Yin, Dong Yin, Mengjin Zhu, Mei Yu, Xinyun Li, Shuhong Zhao, Xiaolei Liu

**Affiliations:** ^1^Key Laboratory of Agricultural Animal Genetics, Breeding and Reproduction, Ministry of Education & College of Animal Science and Technology, Huazhong Agricultural University, Wuhan, China; ^2^Key Laboratory of Swine Genetics and Breeding, Ministry of Agriculture, Huazhong Agricultural University, Wuhan, China

**Keywords:** GWAS, pig, growth traits, ADG, AGE

## Abstract

Improvement of the growth rate is a challenge in the pig industry, the Average Daily Gain (ADG) and Days (AGE) to 100 kg are directly related to growth performance. We performed genome-wide association study (GWAS) and genetic parameters estimation for ADG and AGE using the genomic and phonemic from four breed (Duroc, Yorkshire, Landrace, and Pietrain) populations. All analyses were performed by a multi-loci GWAS model, FarmCPU. The GWAS results of all four breeds indicate that five genome-wide significant SNPs were associated with ADG, and the nearby genomic regions explained 4.08% of the genetic variance and 1.90% of the phenotypic variance, respectively. For AGE, six genome-wide significant SNPs were detected, and the nearby genomic regions explained 8.09% of the genetic variance and 3.52% of phenotypic variance, respectively. In total, nine candidate genes were identified to be associated with growth and metabolism. Among them, *TRIB3* was reported to associate with pig growth, *GRP, TTR, CNR1, GLP1R, BRD2, HCRTR2, SEC11C, and ssc-mir-122* were reported to associate with growth traits in human and mouse. The newly detected candidate genes will advance the understanding of growth related traits and the identification of the novel variants will suggest a potential use in pig genomic breeding programs.

## Introduction

Growth rate is a vital economic trait and significantly affected pig production (Fontanesi et al., 2014; Ding et al., 2018). It is usually measured by Average Daily Gain (ADG), which is average daily weight gain within a certain period as well as Age (AGE) that adjusted to a certain weight. ADG and AGE are directly related to the pig growth and commonly used in pig breeding. Both traits are in moderate heritability and can be efficiently selected by modern breeding techniques (Hoque et al., 2007).

Many growth traits relevant candidate genes were identified since the development of sequencing technology. Up to now, 609 Quantitative Trait Loci (QTLs) were reported that associated with ADG and AGE (Hu et al., 2016). Previous researches have shown a series of candidate genes of ADG and AGE. *XIRP2* and *MC4R* gene were reported that associated with both ADG and residual feed intake (RFI) in pure Duroc population (Onteru et al., 2013; Do et al., 2014). The *SOGA1* gene and *ZFPM2* gene were identified to associate with both ADG and AGE in pure Duroc population (Do et al., 2014; Meng et al., 2017). The *IGSF3* and *IGF2* gene were detected to associated with ADG in an Italian Large White pig population (Van Laere et al., 2003; Fontanesi et al., 2014).

The genetic architectures of growth traits are usually complex and generally controlled by multiple gene. With the developments of cost effective genotyping technology, Genome-wide association study (GWAS) has been widely used for mapping candidate genes of complex traits (Cantor et al., 2010; Korte and Farlow, 2013). Mixed linear model (MLM), which simultaneously incorporates principal components as fixed effects and individual additive effects as random effects, has become one of the most popular models used in GWAS (Yu et al., 2006). Multiple algorithms have been developed to boost both the computational efficiency and statistical power of MLM methods (Kang et al., 2008; Zhou and Stephens, 2012). However, previous studies indicated that the confounding problem decreased the power of MLM for detecting candidate genes that associated with population structure, especially in a population with explicit population structure. Many GWAS analyses for growth traits were carried out in single breed with limited sample size (Ding et al., 2018). Therefore, the candidate genes that contributed to the variation among breeds were always missed. Our previous study introduced FarmCPU method, which is a multiple loci model and split the MLM into separated fixed effect model and random effect model and iteratively uses above two models (Liu et al., 2016). A series of studies in both livestock and plant indicated that FarmCPU detected more candidate genes by solving the confounding problem (Kaler et al., 2017; Kusmec et al., 2017; Meng et al., 2017; Wang et al., 2018).

In this study, GWAS was conducted in a population of 4,865 pigs composed of four pure breeds (Duroc, Yorkshire, Landrace, and Pietrain) for mapping candidate genes underlying ADG and AGE traits. Estimation of genetic parameters, such as heritability was also carried out. The detected candidate genes, potential breeding markers, and the better understanding on genetic architectures of ADG and AGE will benefit breeding programs.

## Materials and Methods

### Animals and Phenotypes

Animals used in this study were from two isolated farms, and composed of four pure breeds, including Duroc, Yorkshire, Landrace, and Pietrain (Supplementary Table S1). ADG and AGE were measured from 70 to 115 kg, and then adjusted to 100 kg. AGE was adjusted to 100 kg using formula below:

$$\begin{array}{l} AGE\;adjusted\;to\;100kg=Measured\;age\\ -\left[\frac{\left(Measured\;weight-100KG\right)}{Correction\;factor}\right] \end{array}$$

where correction factors are different for sire and dam, and the formulas are shown below:

$$\begin{array}{l} Sire:\;Correction\;factor=\frac{Measured\;weight}{Measured\;age}*1.826\\ Dam:\;Correction\;factor=\frac{Measured\;weight}{Measured\;age}*1.715 \end{array}$$

ADG was calculated by following equation:

$$\begin{array}{l} ADG\;adjusted\;to\;100kg=\frac{100\;kg}{AGE\;adjusted\;to\;100\;kg} \end{array}$$

In total, 31,173 phenotypic observations from farm 1 and 24,374 phenotypic observations from farm 2 were recorded. Tail and ear tissue samples were collected and preserved with 75% alcohol and were stored in −20°C freezers.

### Genotyping, Imputation, and Quality Control

Genomic DNA was extracted from frozen collected ear tissue samples using Tecan Freedom EVO NGS workstation and TIANGEN magnetic animal tissue genomic DNA kit. DNA samples with concentration ≥40*ng*/μ*l*, quantity ≥ 1 μ*g*, and passed gel electrophoresis test were used for genotyping. A total of 4,865 DNA samples, including 1,595 samples from farm 1 and 3,270 samples from farm 2 were genotyped by Illumina PorcineSNP50 Bead Chip, which includes 50,697 SNPs, and all SNP markers were mapped to Sus scrofa genome build 11.1 (Ramos et al., 2009). The data in PLINK binary format is available at https://figshare.com/articles/pig-growth-data_zip/7533020.

Missing genotype data were fully imputed by FImpute version 2.2 (Sargolzaei et al., 2014). Data quality control was performed by PLINK and G2P software before and after imputation (Chang et al., 2015; Tang and Liu, 2019). The data was filtered by genotype call rate >0.9, Minor Allele Frequency (MAF) < 0.01, and individuals with SNP marker call rate <0.9 and missing phenotypic records were also removed. The remaining data was used for the subsequent analysis (Supplementary Table S1).

### Estimation of Genetic Parameters

Genetic parameters, including genetic variance, residual variance, and heritability were estimated using Average Information - Restricted Maximum Likelihood algorithm (AI-REML) in an R procedure (Gilmour et al., 1995). The parameters were estimated by three methods, including pedigree-based Best Linear Unbiased Prediction (aBLUP), genomic BLUP (gBLUP), and single-step BLUP (ssBLUP) (Henderson, 1975; VanRaden, 2008; Aguilar et al., 2010). All BLUP models can be written as:

$$\begin{array}{l} y=Xb\;+\;Zu\;+\;e \end{array}$$

where ***y*** is a vector of phenotypic observations; ***b*** and ***u*** represent fixed effects and breeding values, respectively; ***X*** and ***Z*** were design matrices for ***b*** and ***u***, respectively; ***e*** represents the residual error vector with a normal distribution of $e\sim N\left(0,I\sigma_{e}^{2}\right)$, where ***I*** was an identity matrix and $\sigma_{e}^{2}\;$is residual variance. In aBLUP model, $u\sim N\left(0,{A\sigma }_{u}^{2}\right)$, in which $\sigma_{u}^{2}$ is additive genetic variance and ***A*** is an additive genetic relationship matrix that derived from pedigree records; In gBLUP model, ***A*** is replaced by ***G*** and $u\sim N\left(0,{G\sigma }_{u}^{2}\right)$, ***G*** is derived from genomic information and can be constructed by VanRaden method:

$$G\;=\frac{\;M^{\prime }M}{2\;\sum_{i=1}^{m}p_{i}(1-p_{i})}$$

where ***M*** is a ***m*×*n*** standardized genotype matrix, ***m*** is the marker size and ***n*** is the number of genotyped individuals, *p*_*i*_ is the minor allele frequency of the ***i***^***th***^ genetic marker; In ssBLUP method, $u\sim \left(0,{H\sigma }_{u}^{2}\right)$ and the relationship matrix ***H*** is derived from both pedigree records and genomic information simultaneously using the following equation:

$$\begin{array}{l} H\;=\;\left(\begin{array}{cc} A_{11}-A_{12}A_{22}^{-1}A_{21}+A_{12}A_{22}^{-1}GA_{22}^{-1}A_{21} & A_{12}A_{22}^{-1}G\\ GA_{22}^{-1}A_{21} & G+\alpha A_{22} \end{array}\right) \end{array}$$

Individuals are assigned to different groups based on available information. The group with footer ***1*** represents individuals that only have pedigree information and group with footer ***2*** represents individuals that have both pedigree and genomic information. The *A*_11_ and *A*_22_ represent the relationship among individuals within group ***1*** and group ***2***, respectively, *A*_12_ represents the relationship among individuals between group ***1*** and group ***2*** and *A*_21_ is the transpose of *A*_12_, **α** is the ratio for combing ***G*** and *A*_22_ matrix and set to 0.05 in this study.

### Genome-Wide Association Study

Association tests were performed by a multi loci model, FarmCPU (Liu et al., 2016). FarmCPU model iteratively uses fixed effect model and random effect model. The top three columns of principal components, sex, farm effects, and pseudo QTNs (Quantitative Trait Nucleotides) were added as covariates in the fixed effect model for association tests and the model can be written as:

$$\begin{array}{l} y=Pb_{p}+\;M_{t}b_{t}+S_{j}d_{j}+e \end{array}$$

where ***y*** is phenotypic observation vector; ***P*** is a matrix of fixed effects, including top three principal components, sex, and farm effects; ***M***_***t***_ is the genotype matrix of ***t*** pseudo QTNs that used as fixed effects; ***b***_***p***_ and ***b***_***t***_ are the relevant design matrices for ***P*** and ***M***_***t***_, respectively; ***S***_***i***_ is the ***ith*** marker to be tested and ***d***_***j***_ is the corresponding effect; ***e*** is the residual effect vector and $e\sim N(0,I\sigma_{e}^{2})$. Random effect model is used for selecting the most appropriate pseudo QTNs. The model is written as:

$$\begin{array}{l} y=u+e \end{array}$$

where **y** and **e** stay the same as in fixed effect model; **u** is the genetic effect and $u\sim N(0,K\sigma_{u}^{2})$, in which ***K*** is the relationship matrix that defined by pseudo QTNs.

The Bonferroni correction threshold for multiple tests was used for detecting the genome-wide significant SNPs, which defined as α/*K* (α = 0.05 and *K* is the number of SNPs) (Nicodemus et al., 2005).

### Variance Explained by Candidate Regions

Candidate region is defined as the genomic region that located within 1 Mb upstream and downstream of the genome-wide significant SNPs. The proportion of genetic variance and phenotypic variance explained by candidate regions are estimated in a mixed linear model with multiple random effects. The model can be written as:

$$\begin{array}{l} y=Xb\;+\;Z_{i}u_{i}\;+\;e \end{array}$$

where ***y***, ***Xb***, and ***e*** are the same as described in **2.3**. ***u***_***i***_ represents the ***ith*** random effect and $u_{i}\sim N(0,k_{i}\sigma_{ui}^{2})$, in which ***k***_***i***_ and $\sigma_{ui}^{2}$ represent the variance covariance matrix and variance of the ***ith*** random effect, respectively.

In this study, ***k***_**1**_ is a relationship matrix that defined by all SNPs within the candidate regions and ***k***_***2***_ is constructed by the rest of SNPs. The ratio between $\sigma_{u1}^{2}$ and the sum of $\sigma_{u1}^{2}$ and $\sigma_{u2}^{2}$ is defined as the proportion of genetic variance explained by candidate regions. The proportion of phenotypic variance explained by candidate regions can be calculated as the ratio between $\sigma_{u1}^{2}$ and the phenotypic variance. Additionally, the variance explained by randomly selected genomic regions was recognized as null distribution and compared with the variance explained by the candidate regions.

### Identification of Candidate Genes

The candidate genes nearby the genome-wide significant SNPs were identified by Ensembl database using the gene annotation information of Sus scrofa genome version v11.1 (www.ensembl.org/biomart/). The genomic locations were downloaded from https://www.animalgenome.org/pig/ and genes within the candidate region are considered as candidate genes.

## Results

### Summary Information of Phenotype Data, Genotype Data, and Population Structure

Summary statistics of both ADG and AGE were analyzed and shown in Table 1 and Supplementary Table S2. Both traits followed normal distribution and phenotypic distribution is plotted in Supplementary Figure S1. The genotype data of the four breeds includes 4,260 individuals with 47,157 SNPs and SNP density plot of each chromosome is shown in Supplementary Figure S2. Principal component analysis (PCA) was carried out and the scatterplot of the first two principal components are displayed in Supplementary Figures S3, S4.

**Table 1:** Summary statistics of ADG and AGE that adjusted to 100 kg for all breeds.

**Traits**	**Farm**	Mean ± SD^*^	**Median**	CV^*^	**Population size**
ADG	Farm1	0.64 ± 0.035	0.64	0.055	1,567
	Farm2	0.62 ± 0.052	0.62	0.083	2,693
	ALL	0.63 ± 0.047	0.63	0.075	4,260
AGE	Farm1	154.38 ± 8.522	154.07	0.055	1,567
	Farm2	159.25 ± 13.542	158.27	0.085	2,693
	ALL	157.46 ± 12.171	156.33	0.077	4,260

* *SD, Standard Deviation; CV, Coefficient of Variation*.

### Estimation of Genetic Parameters

Genetic variance, residual variance, and heritability of ADG and AGE that adjusted to 100 kg on two farms were estimated by AI-REML using three models for data of all four breeds. Heritability was calculated by dividing genetic variance with the sum of genetic variance and residual variance. All estimated genetic parameters are shown in Table 2. The heritability estimations by the three methods are ranged between 0.408 and 0.562, and 0.444 and 0.572 for ADG and AGE, respectively. The results indicate that the heritabilities of ADG and AGE in two farms are similar and Farm2 has higher phenotypic variation compared with Farm1.

**Table 2:** Estimation of genetic parameters for ADG and AGE that adjusted to 100 kg.

**Trait**	**Farm**	**Models**	Additive genetic variance ± SE^*^	Residual variance ± SE^*^	*h*^***2***^ ± SE^*^
ADG	Farm1	ssBLUP	0.000785 ± 0.0000	0.00105 ± 0.0000	0.428 ± 0.0114
		gBLUP	0.000623 ± 0.0001	0.000611 ± 0.0000	0.505 ± 0.0484
		aBLUP	0.000725 ± 0.0000	0.00105 ± 0.0000	0.408 ± 0.0112
	Farm2	ssBLUP	0.00161 ± 0.0001	0.00134 ± 0.0000	0.544 ± 0.0134
		gBLUP	0.00146 ± 0.0001	0.00114 ± 0.0001	0.562 ± 0.0329
		aBLUP	0.00136 ± 0.0001	0.00138 ± 0.0000	0.496 ± 0.0139
AGE	Farm1	ssBLUP	62.868 ± 2.1715	71.557 ± 1.2309	0.468 ± 0.0122
		gBLUP	34.965 ± 4.6381	35.397 ± 2.6717	0.497 ± 0.0486
		aBLUP	57.625 ± 1.9962	72.181 ± 1.2182	0.444 ± 0.0120
	Farm2	ssBLUP	131.227 ± 4.5365	98.340 ± 2.2884	0.572 ± 0.0134
		gBLUP	100.574 ± 9.4161	81.417 ± 4.2342	0.553 ± 0.0331
		aBLUP	109.280 ± 3.9940	102.275 ± 2.3317	0.517 ± 0.0141

* *SE, Standard Error*.

### GWAS Results of ADG and AGE for All Breeds

**Figure 1:**
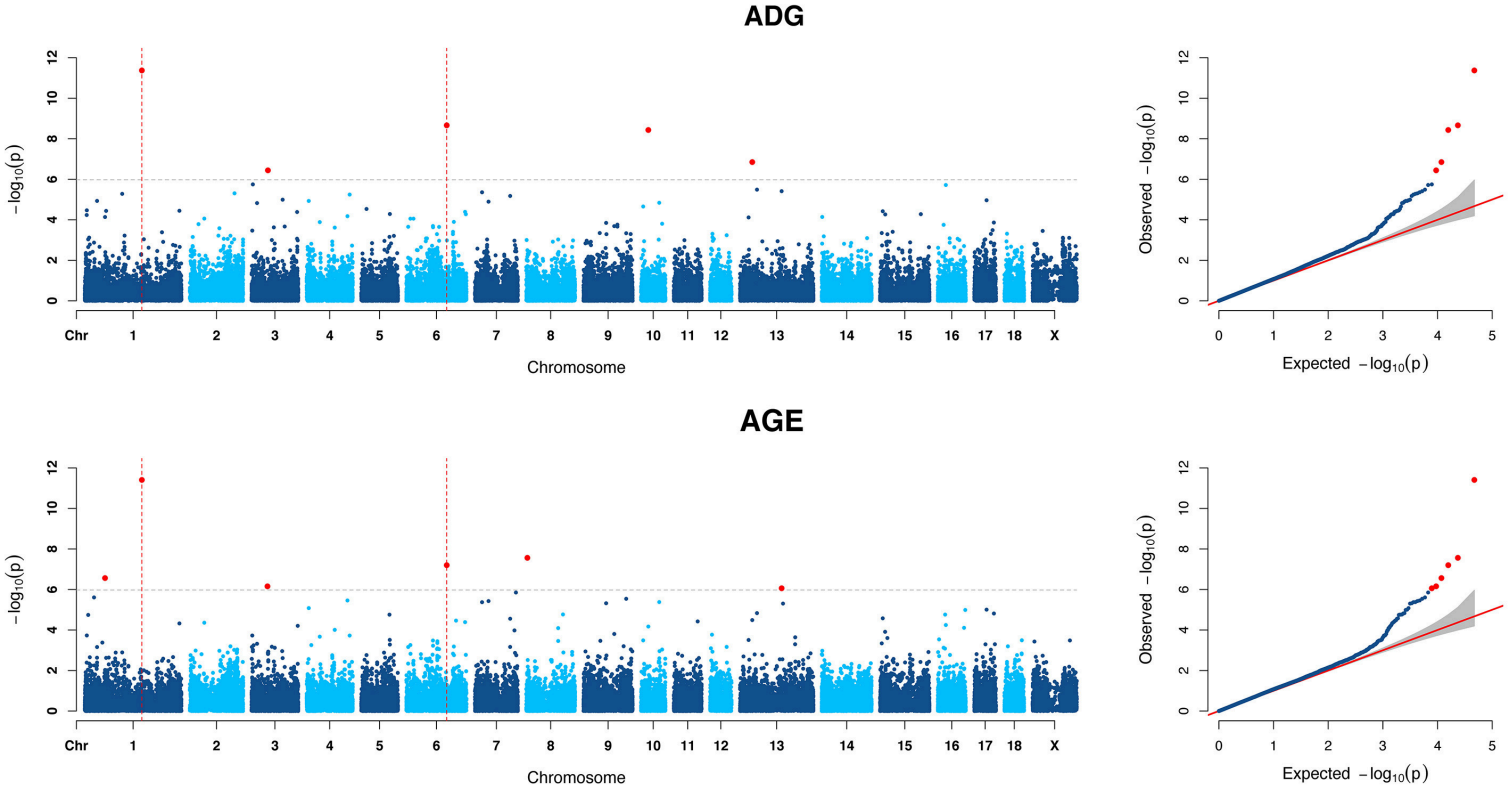
Manhattan plots and Quantile-Quantile (QQ) plots of both ADG and AGE traits for all breeds. The points of genome-wide significant SNPs are colored in red. There are two SNPs that detected in both traits are marked with dotted lines.

For ADG trait, 5 genome-wide significant SNPs were detected and the candidate regions are located on chromosome 1, 3, 6, 10, and 13 (Figure 1). Linkage disequilibrium (LD) analysis was conducted using pooling data of the four breeds and the LD decay is shown in Supplementary Figure S5. The results indicate that LD decay tends to be stable when the distance is 1Mb. Therefore, genes that located within 1 Mb nearby the significant SNPs are defined as candidate genes. The detailed information of top ten significant SNPs, including SNP ID, Chromosome (Chr), Physical positions, *P*-value, and candidate genes are provided in Table 3.

**Table 3:** Summary information of top 10 significant SNPs for ADG trait.

**SNP ID**	**Chr**	**Physical position (bp)**	***P*-value**	**Candidate genes**
WU_10.2_1_179575045	1	161,987,727	4.24E-12	*GRP, SEC11C*
Affx-114707063	6	114,632,185	2.17E-09	*TTR*
WU_10.2_10_25384240	10	20,774,539	3.70E-09	*CAPN2*
DRGA0012293	13	34,889,004	1.42E-07	*TNNC1*
WU_10.2_3_47794141	3	46,520,543	3.62E-07	*STARD7*
WU_10.2_3_4365368	3	3,714,738	1.79E-06	*ACTB*
ASGA0072627	16	22,729,510	1.92E-06	*NIPBL*
MARC0071621	13	48,304,723	3.24E-06	*SUCLG2*
MARC0005049	13	118,274,633	3.87E-06	*ACTL6A*
H3GA0020255	7	20,299,952	4.39E-06	*SCGN*

We consider the genomic region that located within 1 Mb distance on either side of the significant SNPs as candidate region. For ADG, the five candidate regions explained 4.08 and 1.90% of the genetic and phenotypic variance, respectively. In contrast, a randomly selected five regions across the whole genome repeated for 50 times, on average, five randomly selected genomic regions could only explain 0.663 and 0.311% of the genetic and phenotypic variance, respectively. The detailed information is shown in Table 4.

**Table 4:** The proportion of genetic variance and phenotypic variance explained by candidate regions.

**Trait**	**No. of randomly selected regions**	**Vg ± SE**	**Vp ± SE**	**No. candidate regions**	**Vg**	**Vp**
ADG	5	0.663 ± 0.0013%	0.311 ± 0.0006%	5	4.08%	1.90%
AGE	6	0.669 ± 0.0010%	0.304 ± 0.0005%	6	8.09%	3.52%

*Vg, Proportion of Genetic Variance; Vp, Proportion of Phenotypic Variance*.

Six genome-wide significant SNPs were detected in AGE trait and detailed information of top ten significant SNPs is shown in Table 5. These candidate genomic regions explained 8.09 and 3.52% of the total genetic and phenotypic variance for AGE, respectively. In comparison, a randomly selected six regions across the whole genome repeated for 50 times, on average, can only explained 0.669 and 0.304% of the genetic variance and phenotypic variance (Table 4), respectively. Due to the genetic correlation between ADG and AGE, two SNPs that located on chromosome 1 and chromosome 6 are detected in both traits and marked with dotted line in Figure 1.

**Table 5:** Summary information of top ten significant SNPs for AGE trait.

**SNP ID**	**Chr**	**Physical position (bp)**	***P*-value**	**Candidate genes**
WU_10.2_1_179575045	1	161,987,727	3.91E-12	*GRP*
WU_10.2_8_3769689	8	3,371,469	2.74E-08	*AFAP1*
Affx-114707063	6	114,632,185	6.32E-08	*TTR*
MARC0065740	1	57,399,350	2.73E-07	*CNR1*
WU_10.2_3_47139500	3	45,234,651	6.99E-07	*ACOXL*
MARC0005049	13	118, 274, 633	8.70E-07	*DNAJC19*
WU_10.2_7_123699934	7	116,483,173	1.41E-06	*CLMN*
WU_10.2_1_29330685	1	26,181,399	2.47E-06	*PEX7*
MARC0010417	9	121,197,647	2.88E-06	*SOAT1*
ASGA0022397	4	115,787,208	3.49E-06	*COL11A1*

### GWAS Results of ADG and AGE for Single Breed

**Figure 2:**
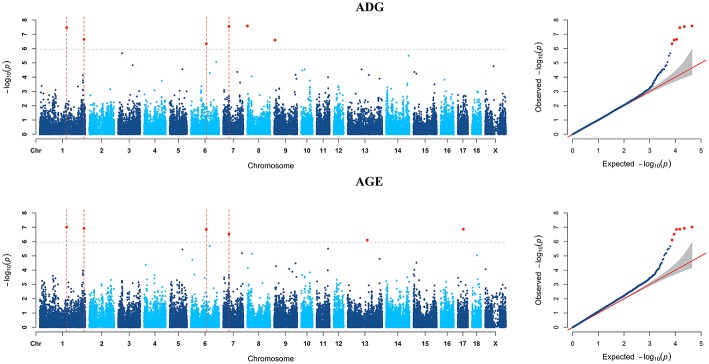
Manhattan plots and Quantile-Quantile (QQ) plots for both ADG and AGE traits of Duroc breed. The signals of genome-wide significant SNPs are colored in red. There are four SNPs that detected in both traits are marked with dotted lines.

**Figure 3:**
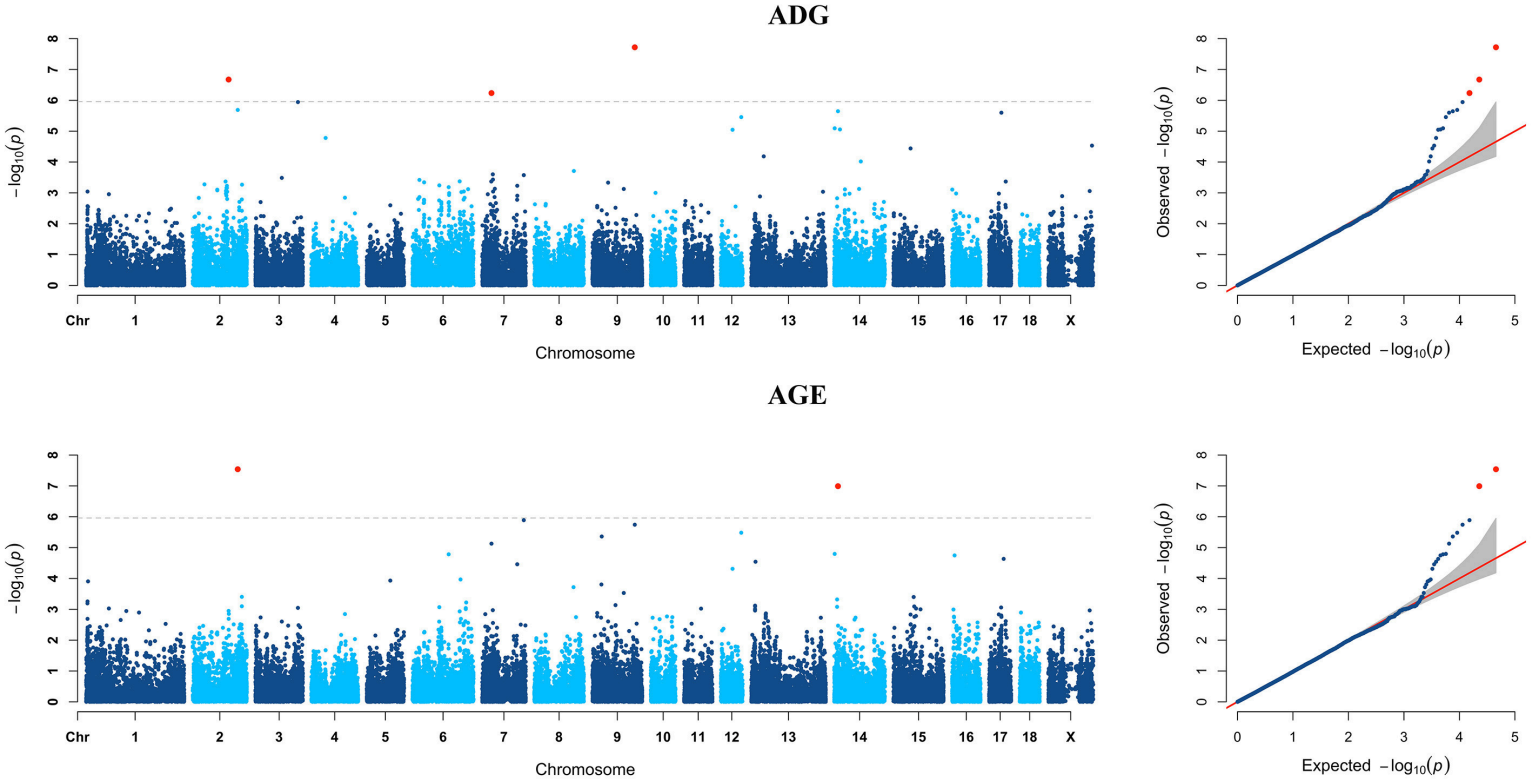
Manhattan plots and Quantile-Quantile (QQ) plots for both ADG and AGE traits of Landrace breed. The signals of genome-wide significant SNPs are colored in red.

**Figure 4:**
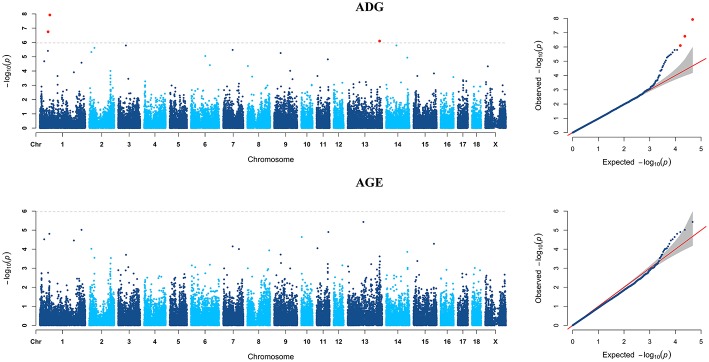
Manhattan plots and Quantile-Quantile (QQ) plots for both ADG and AGE traits of Yorkshire breed. The signals of genome-wide significant SNPs are colored in red.

**Figure 5:**
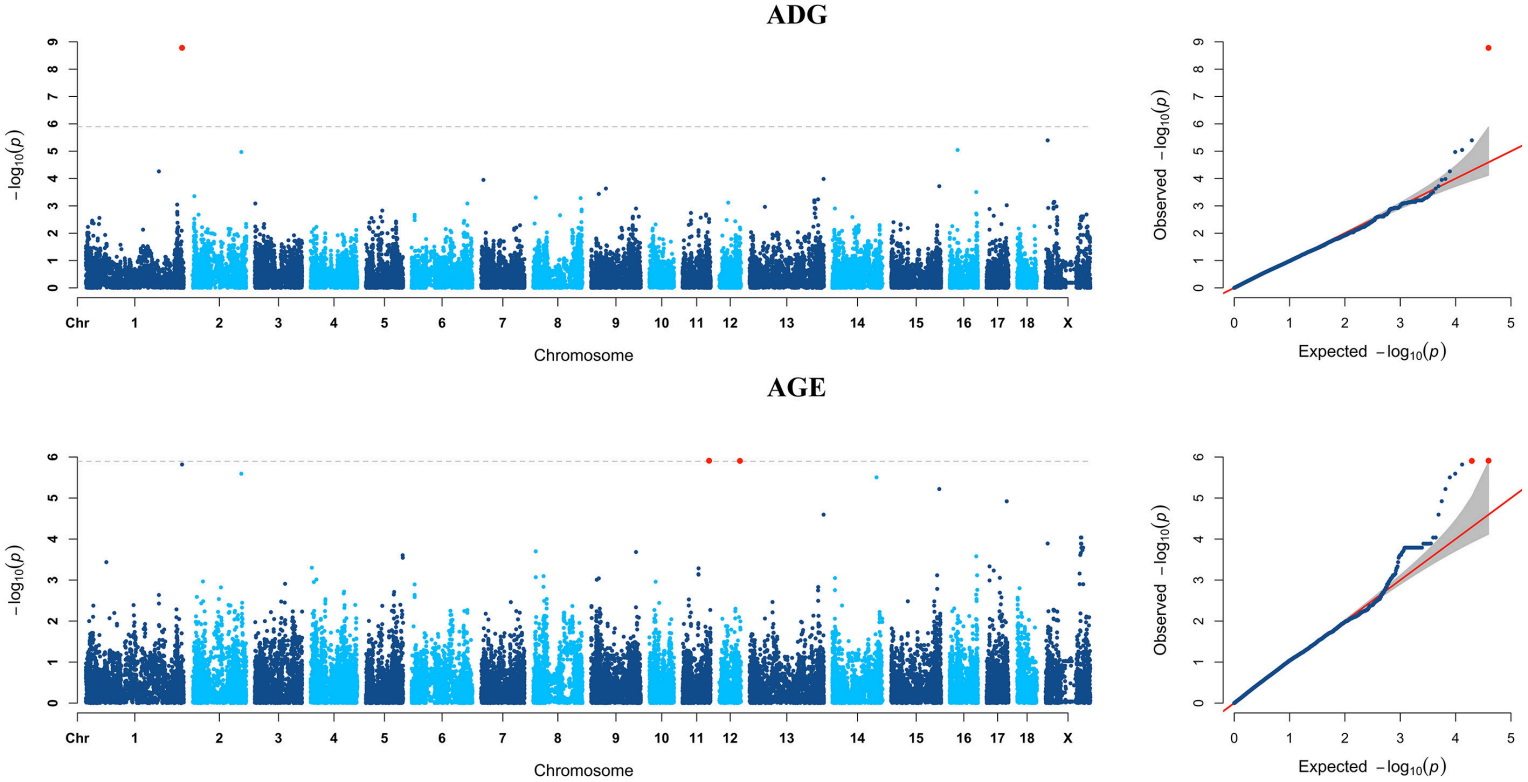
Manhattan plots and Quantile-Quantile (QQ) plots for both ADG and AGE traits of Pietrain breed. The signals of genome-wide significant SNPs are colored in red.

GWAS analyzes for single breed were also carried out. Six genome-wide significant SNPs were detected for both ADG and AGE traits by Duroc population (Figure 2, Supplementary Table S3). Among the significant SNPs detected by Duroc population, there are two signals are consistent with the genome-wide significant SNPs detected by the data of all breeds. GWAS results of Landrace, Yorkshire, and Pietrain are displayed in Figures 3–5. The detailed information of top ten significant SNPs detected by the single breed population are shown in Supplementary Tables S4–S6.

## Discussion

Most target economic traits in livestock are quantitative traits and usually with a complex genetic architectures. Therefore, revealing the candidate genes underlying these traits is always the attractive area of research in livestock genetics and breeding (Goddard and Hayes, 2009; Hou and Zhao, 2013). Since the GWAS research on age-related macular degeneration of the retina was published, GWAS has become one of the most popular method for identifying candidate genes that associated with target traits (Klein et al., 2005). With the developments of commercialized high density SNP Chips for multiple livestock species, the GWAS has been widely used in livestock research and breeding (Zhang et al., 2012; Wang et al., 2017). In this study, the ADG and AGE traits of pigs from two separated farms were obtained. A population of 5,000 individuals of four pure breeds were genotyped by 50K SNP Chip. For all breeds, a total of 4,260 individuals with 47,157 SNPs were used for association tests using the FarmCPU method, which is a multi-loci GWAS model. For both ADG and AGE, nine genome-wide significant SNPs were detected using data of all four breeds, including two common detected signals. The candidate regions were evaluated in a mixed linear model with multiple random effects for the variance contribution and explained about 4–8% of the genetic variance and 2–3% of the phenotypic variance.

ADG and AGE are perfectly correlated after take the log transformation. A trait and its inverse values or log transformation values take different assumptions for the genetic architectures. For a trait with complex genetic architecture, common used hypothesis assume that the effects of genes are linear. However, there exist some restrictions of the phenotype and some genes that don't directly affect the trait may also restrict its maximum or minimum. The effects of genes are not always equally contributed to every level of the trait and the effects of genes are non-linear sometimes. The linear assumption fits perfectly near the average value of traits, but it may lose its fitness near the boundary of the trait. The non-linear assumption utilizes the information near boundary more effectively. Due to the inverse relationship of ADG and AGE, the analyzes on both traits seem like to use different modeling way to detect the candidate genes underlying the pig growth rate. From the results, we can also see that ADG and AGE have some common detected signals and some disagreements were also found as well.

The heritabilities of both ADG and AGE traits were estimated by three models including aBLUP, gBLUP and ssBLUP using the pooling data of the four breeds. The heritabilities of ADG and AGE traits are 0.41~0.56 and 0.44~0.57, respectively. Previous research also reported that ADG is a moderate heritability trait and its heritability is approximately 0.45~0.49 in a Duroc population (Gilbert et al., 2007; Hoque et al., 2009). Comparing the heritabilities that estimated by three models, the standard error of gBLUP model is higher than the other two models because of the small genotyped population size (Gutierrez et al., 2018). The ssBLUP method utilizes the pedigree and genomic information simultaneously and the estimated genetic parameters are theoretically more accurate (Meuwissen et al., 2016).

Using data of all breeds, there are two SNPs detected in both ADG and AGE traits, which are WU_10.2_1_179575045 on chromosome 1 and Affx-114707063 on chromosome 6. *GRP, SEC11C (SEC11 homolog C)*, and *ssc-mir-122* were identified in the nearby candidate regions on chromosome 1. *GRP* is a regulatory neuropeptide that stimulates gastric G cells to release gastrin and regulate gastric acid secretion and intestinal movement, which affects the food intake and may lead to anorexia, bulimia, and obesity if lacked of the genes (Merali et al., 1999). *SEC11C* is a homolog of *SEC11*, which is the only essential factor for signal peptide processing and plays an important role in protein processing, localization, and secretion. The lack of *SEC11* will cause serious growth defects (Bohni et al., 1988). The *ssc-mir-122* is a highly evolutionarily conserved miRNA and it is an important regulator of lipid metabolism (Esau et al., 2006). A previous experiment shows that the weight and cholesterol levels are increased with the decreased expression of *ssc-mir-122* by feeding high-level cholesterol feed to mini pigs (Cirera et al., 2010). Previous published researches also verified that *MC4R* and *GTF3C5* that located on chromosome 1, are candidate genes for growth traits (Onteru et al., 2013; Quan et al., 2018). For Affx-114707063 SNP on chromosome 6, the identified nearby candidate gene is *TTR*, which is the main carrier of thyroid hormone (*T4*) and plays an important role on growth and energy metabolism (Richardson et al., 2007). The concentration of *TTR* in the blood can be used to assess nutritional status. Because of its short half-life, the concentration can better reflect the nutritional levels of recent dietary intake (Shenkin, 2006). Some researches verified that *PGM1* (Phosphoglucomutase-1) and *FTO* (Fat mass and obesity-associated protein) that located on chromosome 6 significantly affected muscle development and obesity, respectively (Onteru et al., 2013; Fontanesi et al., 2014).

The *CNR1* gene located on chromosome 1, which next to the significant SNP MARC0065740, is an appetite-related gene and appetite is one of the important factors influencing growth rate. *CNR1* is the major receptor of anandamide that inhibits gastrointestinal activity (Mathison et al., 2004). The *CNR1* receptor inverse agonist rimonabant has been proved to reduce the food intake in both human and mouse. In addition, when the stomach is in contraction, which is a relatively active state, *CNR1* will promote the release of ghrelin, which increases the palatability of food, and it is the origin of the appetite stimulating effect (De Luca et al., 2012).

The SNP density limits the interpretation of GWAS hits due to the different LD between SNP and QTLs in various breeds. Therefore, GWAS was carried out on data of four breeds separately as well. Two signals that detected in Duroc population were consisted with GWAS results of all breeds and explained the variability of the traits among breeds and within Duroc breed. In addition, several genome-wide significant SNPs were detected and contributed to the variability of traits within breeds. For Duroc breed, the *GLP1R* gene is identified nearby the genome-wide significant SNP MARC0015804. It is a member of the glucagon receptor family and controls the blood sugar level (Brubaker and Drucker, 2002), in addition, it is also expressed in the brain and has a role in appetite control (Kinzig et al., 2002). The *TRIB3* gene, which is identified nearby the M1GA0027226 marker on chromosome 17, was reported to be associated with meat production traits in Italian heavy pigs (Fontanesi et al., 2010). For Landrace breed, *BRD2* and *HCRTR2* genes were identified nearby M1GA0027226 SNP on chromosome 7. The knockout of *BRD2* gene will cause obesity in mouse and *HCRTR2* is a kind of orexin receptor, which plays a vital important role in feeding behavior and balance of energy metabolism (Spinazzi et al., 2006; Belkina and Denis, 2012).

## Conclusions

In this study, we performed GWAS on approximately 5,000 purebred pigs that composed of four breeds from two separated farms. All samples were genotyped by 50K SNP Chip. GWAS was performed on ADG and AGE that adjusted to 100 kg using FarmCPU model. A total of 27 genome-wide significant SNPs were detected and two of them were detected in both traits using the data of all breeds and Duroc breed. Nine candidate genes were detected within 1 Mb nearby the genome-wide significant SNPs. Among them, *TRIB3* on chromosome 17 was reported to be associated with meat production traits in pigs; *GRP, CNR1, SEC11C, and ssc-mir-122* on chromosome 1, *TTR* on chromosome 6 and *GLP1R, BRD2, and HCRTR2* gene on chromosome 7 were reported to be associated with growth traits in human and mouse. The newly detected significant SNPs and newly identified candidate genes in this study can be applied to pig breeding and the information could be also incorporated in genomic selection for ADG and AGE traits to achieve faster growth.

## Ethics Statement

This study was carried out in accordance with the guidelines of the science ethics committee of the Huazhong Agricultural University (HZAU). The science ethics committee of the HZAU approved all the animal experiments that conducted in this study.

## Author Contributions

XiaL, SZ, and XinL conceived the study. ZT, LY, and JX wrote the codes and did the data analysis. XiaL and ZT drafted the manuscript and all authors contributed to finalizing the writing.

### Conflict of Interest Statement

The authors declare that the research was conducted in the absence of any commercial or financial relationships that could be construed as a potential conflict of interest.
