# Supplementary material for: Genome-Wide Association Study Reveals Candidate Genes for Growth Relevant Traits in Pigs
Source: Front Genet. 2019 Apr 5;10:302. doi: 10.3389/fgene.2019.00302 (PMC6459934; doi:10.3389/fgene.2019.00302)
Supplement: Supplementary file 1 [file Data_Sheet_1.docx]

Supplementary Material

# Phenotypic distribution of ADG and AGE traits for data of four breeds

**Supplementary Figure S1.** Phenotypic distribution of ADG and AGE that adjusted to 100kg for data of four breeds. The Shapiro-Wilk test showed that both traits followed normal distributions well (p-value < 1e-7).

# SNP density across the genome for data of four breeds

**Supplementary Figure S2.** SNP density on different chromosomes for data of four breeds. The whole genome was divided into 1 Mb windows and marked with different colors based on the marker density of the window.

# Plot of first two principal components for data of four breeds

**Supplementary Figure S3.** The first two principal components are plotted to display the population structure for data of all breeds. Duroc (D), Landrace (L), Pietrain (P) , and Yorkshire (Y) breeds are clustered separately.

**4 Plot of the first two principal components for each breed**


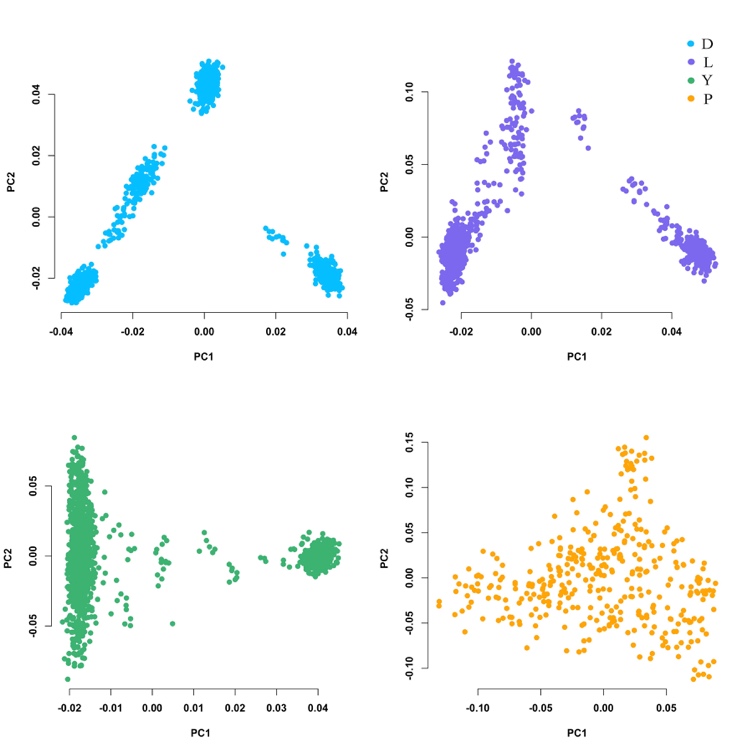


**Supplementary Figure S4.** The first two principal components are plotted to display the population structure within each breed. Duroc (D), Landrace (L), Yorkshire (Y) and Pietrain (P), breeds are colored in blue, violet, green, and orange, respectively.

# 5 Linkage Disequilibrium Decay

**Supplementary Figure S5.** The linkage disequilibrium decay in populations of Duroc, Landrace, Pietrain, and Yorkshire.

**6 The Data information for all GWAS analyzes**

| Breed | Sample size (Farm1) | Sample size (Farm2) | Sample and marker size after data quality control |
| --- | --- | --- | --- |
| Duroc | 442 | 854 | 1,296 samples, 44,323 SNPs |
| Landrace | 321 | 788 | 1,109 samples, 45,561 SNPs |
| Yorkshire | 431 | 1,051 | 1,482 samples, 46,592 SNPs |
| Pietrain | 373 | - | 373 samples, 39,301 SNPs |
| All breeds | 1,567 | 2,693 | 4,260 samples, 47,157 SNPs |

**Supplementary Table S1**. Summary description of the data used for GWAS analyzes on all breeds and single breed.

**7 Summary statistics of phenotype data**

| Traits | Breed | Mean ± SD | Median | CV | Population Size |
| --- | --- | --- | --- | --- | --- |
| ADG | Duroc | 0.64±0.052 | 0.64 | 0.082 | 1,296 |
| AGE | Duroc | 155.18±13.198 | 154.13 | 0.085 | 1,296 |
| ADG | Landrace | 0.64±0.044 | 0.74 | 0.069 | 1,109 |
| AGE | Landrace | 155.43±10.921 | 154.55 | 0.070 | 1,109 |
| ADG | Yorkshire | 0.61 ±0.040 | 0.61 | 0.065 | 1,482 |
| AGE | Yorkshire | 162.78±11.024 | 161.55 | 0.068 | 1,482 |
| ADG | Pietrain | 0.66±0.032 | 0.66 | 0.049 | 373 |
| AGE | Pietrain | 150.30 ±7.488 | 149.76 | 0.050 | 373 |

## *SD, Standard Deviation; CV, Coefficient of Variation.

**Supplementary Table S2**. Summary statistics of ADG and AGE traits for four breeds.

**8 Information of top ten significant SNPs detected in Duroc breed**

| Trait | SNP ID | Chr | Physical Position (bp) | P-value |
| --- | --- | --- | --- | --- |
| ADG | WU_10.2_8_514315 | 8 | 1,017,485 | 2.62E-08 |
| ADG | MARC0015804 | 7 | 35,436,469 | 2.86E-08 |
| ADG | WU_10.2_1_179575045 | 1 | 161,987,727 | 3.45E-08 |
| ADG | WU_10.2_1_301067654 | 1 | 267,065,013 | 2.28E-07 |
| ADG | H3GA0026163 | 9 | 5,274,140 | 2.56E-07 |
| ADG | WU_10.2_6_85968524 | 6 | 92,897,940 | 4.65E-07 |
| ADG | SIRI0001150 | 3 | 23,433,950 | 2.15E-06 |
| ADG | WU_10.2_14_147424746 | 14 | 135,705,097 | 3.18E-06 |
| ADG | ALGA0110546 | 6 | 152,590,134 | 8.79E-06 |
| ADG | CASI0004604 | 3 | 87,48,969 | 1.47E-05 |
| AGE | WU_10.2_1_179575045 | 1 | 161,987,727 | 9.74E-08 |
| AGE | WU_10.2_1_301067654 | 1 | 267,065,013 | 1.19E-07 |
| AGE | MARC0074465 | 17 | 34,023,358 | 1.37E-07 |
| AGE | MARC0020820 | 6 | 93,010,249 | 1.42E-07 |
| AGE | MARC0015804 | 7 | 35,436,469 | 3.04E-07 |
| AGE | MARC0005049 | 13 | 118,274,633 | 7.94E-07 |
| AGE | Affx-114707063 | 6 | 114,632,185 | 2.13E-06 |
| AGE | WU_10.2_11_76416142 | 11 | 69,146,378 | 3.13E-06 |
| AGE | 5_80200432 | 5 | 77,253,445 | 3.57E-06 |
| AGE | WU_10.2_7_120450165 | 7 | 113,847,482 | 6.52E-06 |

**Supplementary Table S3**. The summary information of top ten significant SNPs detected in Duroc breed, including SNP ID, Chromosome (Chr), Physical position, and P-value.

**9 Information of top ten significant SNPs detected in Landrace breed**

| Trait | SNP ID | Chr | Physical Position (bp) | P-value |
| --- | --- | --- | --- | --- |
| ADG | WU_10.2_9_130179733 | 9 | 118,528,867 | 1.91E-08 |
| ADG | DRGA0003223 | 2 | 100,397,283 | 2.12E-07 |
| ADG | M1GA0027226 | 7 | 26,125,115 | 5.82E-07 |
| ADG | WU_10.2_3_127106026 | 3 | 119,031,289 | 1.14E-06 |
| ADG | MARC0063557 | 2 | 125,946,476 | 2.05E-06 |
| ADG | ASGA0093295 | 14 | 10,420,453 | 2.26E-06 |
| ADG | MARC0052026 | 17 | 34,537,905 | 2.52E-06 |
| ADG | WU_10.2_12_59543325 | 12 | 56,889,946 | 3.50E-06 |
| ADG | WU_10.2_14_1442014 | 14 | 1,186,857 | 8.07E-06 |
| ADG | H3GA0039144 | 14 | 15,984,992 | 8.79E-06 |
| AGE | MARC0063557 | 2 | 125,946,476 | 2.88E-08 |
| AGE | ASGA0093295 | 14 | 10,420,453 | 1.02E-07 |
| AGE | WU_10.2_7_123699934 | 7 | 116,483,173 | 1.29E-06 |
| AGE | WU_10.2_9_130179733 | 9 | 118,528,867 | 1.81E-06 |
| AGE | WU_10.2_12_59543325 | 12 | 56,889,946 | 3.29E-06 |
| AGE | MARC0022399 | 9 | 26,111,818 | 4.36E-06 |
| AGE | M1GA0027226 | 7 | 26,125,115 | 7.44E-06 |
| AGE | WU_10.2_14_1442014 | 14 | 1,186,857 | 1.60E-05 |
| AGE | ASGA0085547 | 6 | 101,040,019 | 1.65E-05 |
| AGE | WU_10.2_16_7290867 | 16 | 6,878,698 | 1.79E-05 |

**Supplementary Table S4**. The summary information of top ten significant SNPs detected in Landrace breed, including SNP ID, Chromosome (Chr), Physical position, and P-value.

**10 Information of top ten significant SNPs detected in Yorkshire breed**

| Trait | SNP ID | Chr | Physical Position (bp) | P-value |
| --- | --- | --- | --- | --- |
| ADG | DRGA0001020 | 1 | 6,033,8211 | 1.19E-08 |
| ADG | ASGA0002688 | 1 | 49,800,696 | 1.79E-07 |
| ADG | MARC0094534 | 13 | 192,881,107 | 7.99E-07 |
| ADG | MARC0009517 | 14 | 62,484,909 | 1.62E-06 |
| ADG | WU_10.2_3_47794141 | 3 | 46,520,543 | 1.63E-06 |
| ADG | ALGA0012928 | 2 | 31,736,298 | 2.41E-06 |
| ADG | M1GA0010442 | 7 | 57,618,064 | 3.32E-06 |
| ADG | DRGA0000827 | 1 | 48,698,471 | 3.90E-06 |
| ADG | ASGA0009202 | 2 | 13,250,465 | 4.69E-06 |
| ADG | H3GA0026998 | 9 | 39,133,616 | 5.52E-06 |
| AGE | ALGA0071568 | 13 | 94,975,803 | 3.69E-06 |
| AGE | WU_10.2_1_282594005 | 1 | 251,781,867 | 9.59E-06 |
| AGE | MARC0085875 | 11 | 71,100,401 | 1.25E-05 |
| AGE | MARC0065740 | 1 | 57,399,350 | 1.55E-05 |
| AGE | WU_10.2_10_5153484 | 10 | 3,336,483 | 2.28E-05 |
| AGE | DIAS0002980 | 1 | 26,316,436 | 3.01E-05 |
| AGE | DRGA0001918 | 1 | 205,598,064 | 3.48E-05 |
| AGE | ALGA0087312 | 15 | 123,561,274 | 5.19E-05 |
| AGE | M1GA0010442 | 7 | 57,618,064 | 7.10E-05 |
| AGE | MARC0085235 | 11 | 5,181,576 | 8.87E-05 |

**Supplementary Table S5**. The summary information of top ten significant SNPs detected in Yorkshire breed, including SNP ID, Chromosome (Chr), Physical position, and P-value.

**11 Information of top ten significant SNPs detected in Pietrain breed**

| Trait | SNP ID | Chr | Physical Position (bp) | P-value |
| --- | --- | --- | --- | --- |
| ADG | H3GA0005125 | 1 | 26,952,7781 | 1.66E-09 |
| ADG | WU_10.2_X_6278881 | X | 6,031,723 | 4.02E-06 |
| ADG | WU_10.2_16_23318916 | 16 | 22,170,407 | 9.03E-06 |
| ADG | CASI0010120 | 2 | 137,555,946 | 1.07E-05 |
| ADG | MARC0037014 | 1 | 204,613,927 | 5.48E-05 |
| ADG | WU_10.2_13_217396548 | 13 | 207,465,437 | 1.04E-04 |
| ADG | WU_10.2_7_6889468 | 7 | 6,675,175 | 1.04E-04 |
| ADG | ASGA0095426 | 15 | 134,931,205 | 1.12E-04 |
| ADG | ASGA0104824 | 9 | 41,542,118 | 1.91E-04 |
| ADG | H3GA0047277 | 16 | 74,541,662 | 2.32E-04 |
| AGE | H3GA0032629 | 11 | 74,441,326 | 3.13E-04 |
| AGE | WU_10.2_12_60830299 | 12 | 57,908,988 | 1.24E-06 |
| AGE | H3GA0005125 | 1 | 269,527,781 | 1.52E-06 |
| AGE | CASI0010120 | 2 | 137,555,946 | 2.55E-06 |
| AGE | WU_10.2_14_134915667 | 14 | 123,673,077 | 3.13E-06 |
| AGE | ASGA0095426 | 15 | 134,931,205 | 6.03E-06 |
| AGE | WU_10.2_17_63677403 | 17 | 56,617,580 | 1.20E-05 |
| AGE | WU_10.2_13_217396548 | 13 | 207,465,437 | 2.54E-05 |
| AGE | WU_10.2_X_112286406 | X | 99,105,392 | 9.18E-05 |
| AGE | WU_10.2_X_115293941 | X | 100,530,998 | 9.18E-05 |

**Supplementary Table S6**. The summary information of top ten significant SNPs detected in Pietrain breed, including SNP ID, Chromosome (Chr), Physical position, and P-value.
